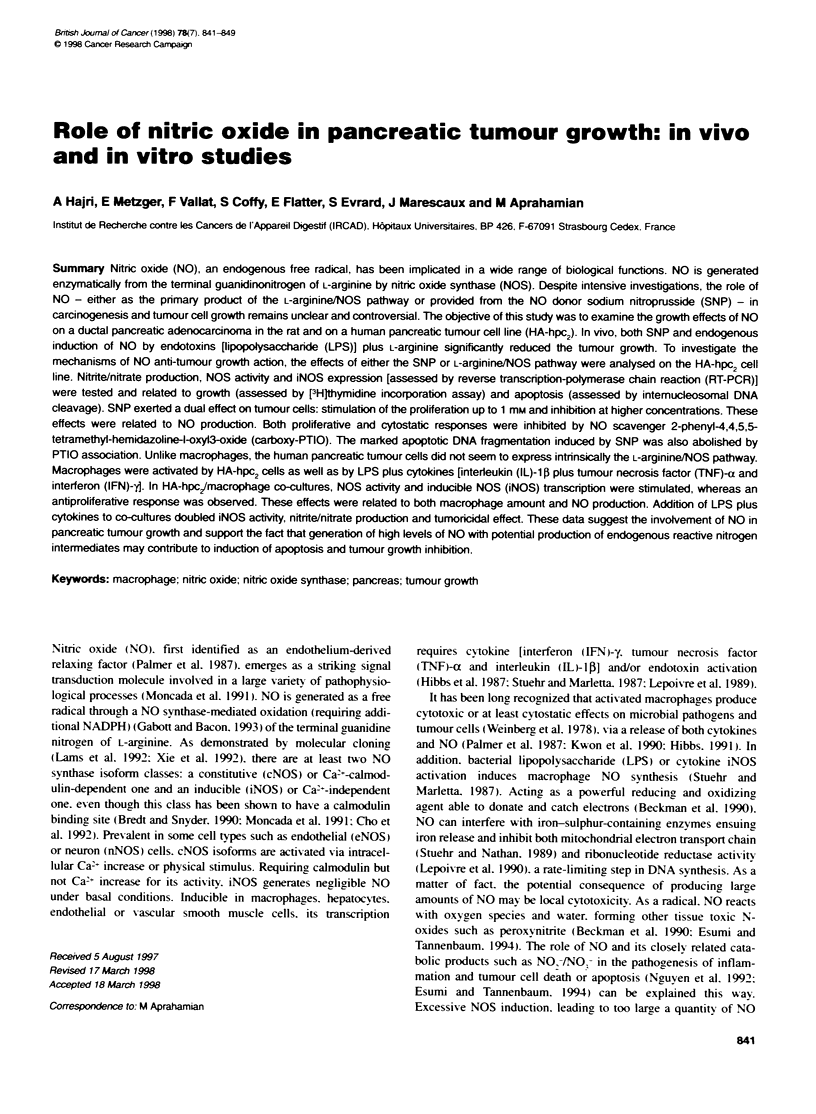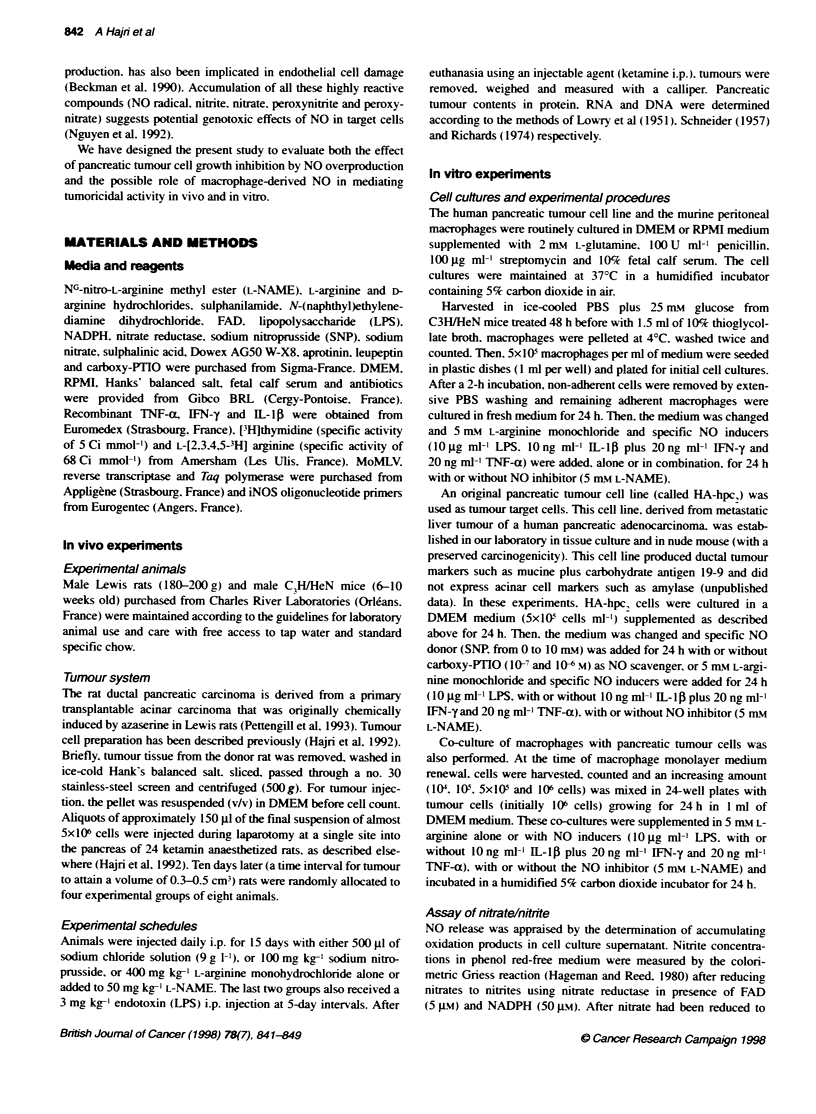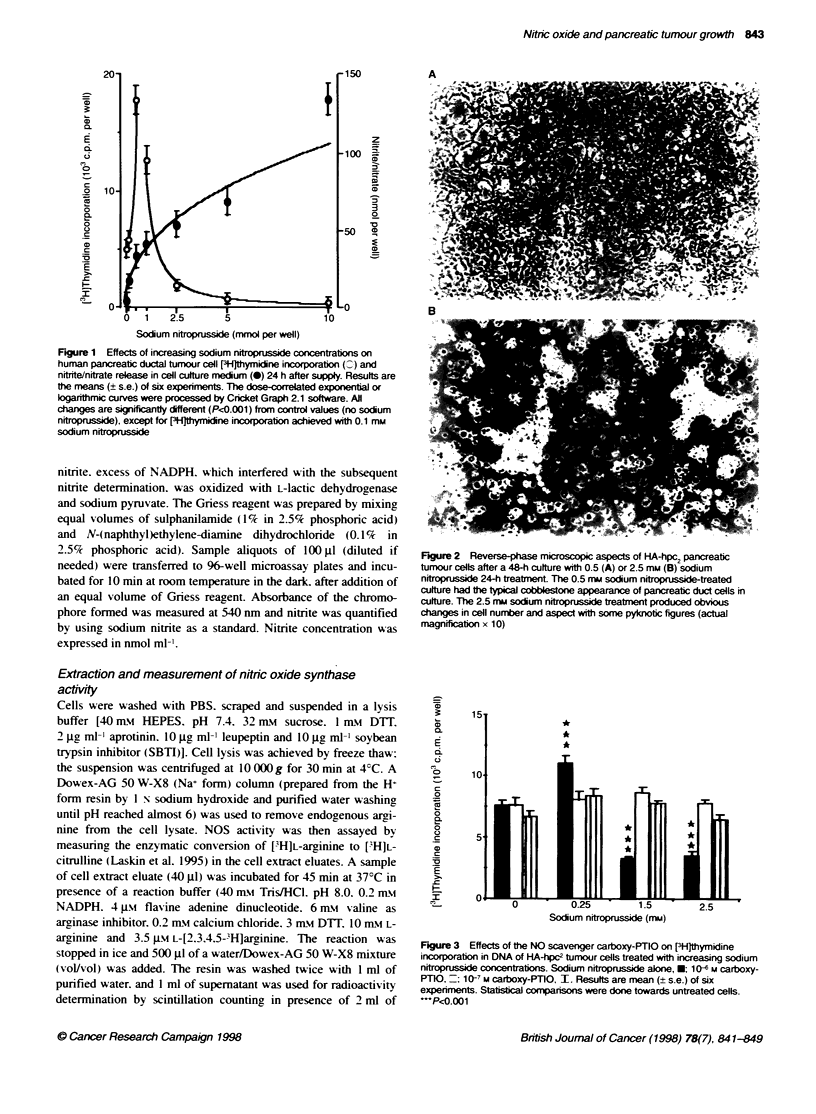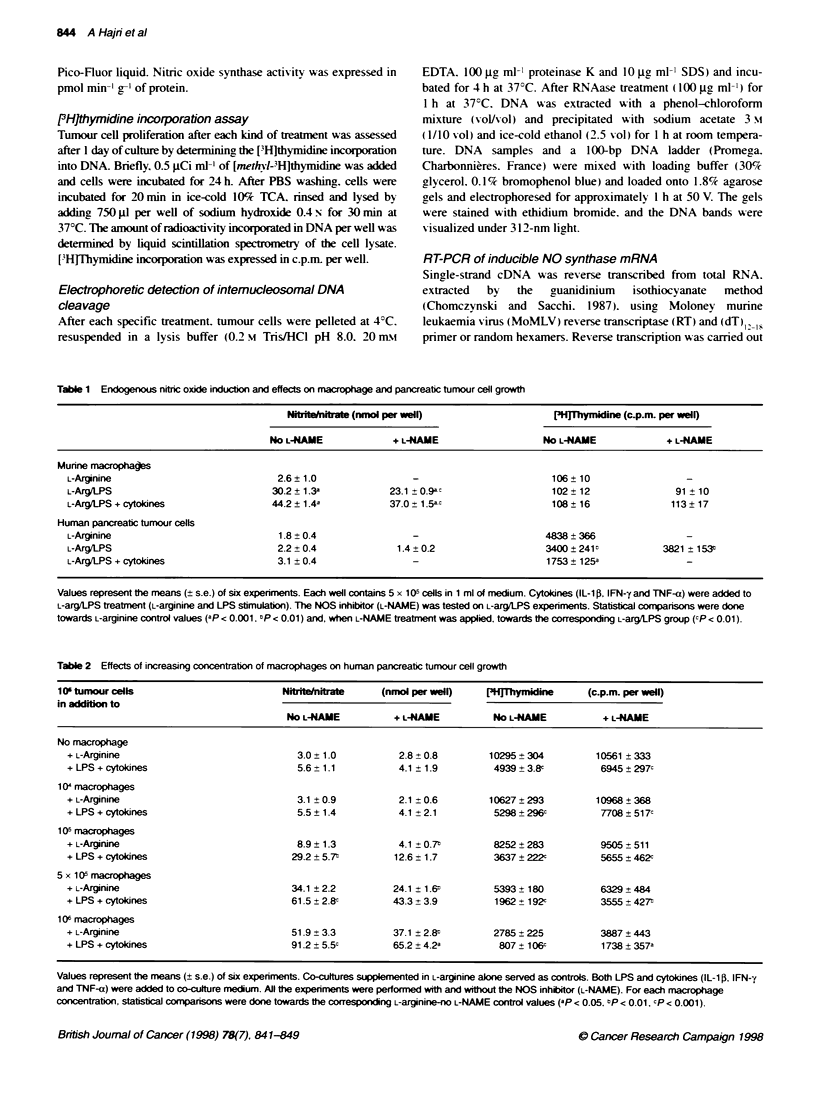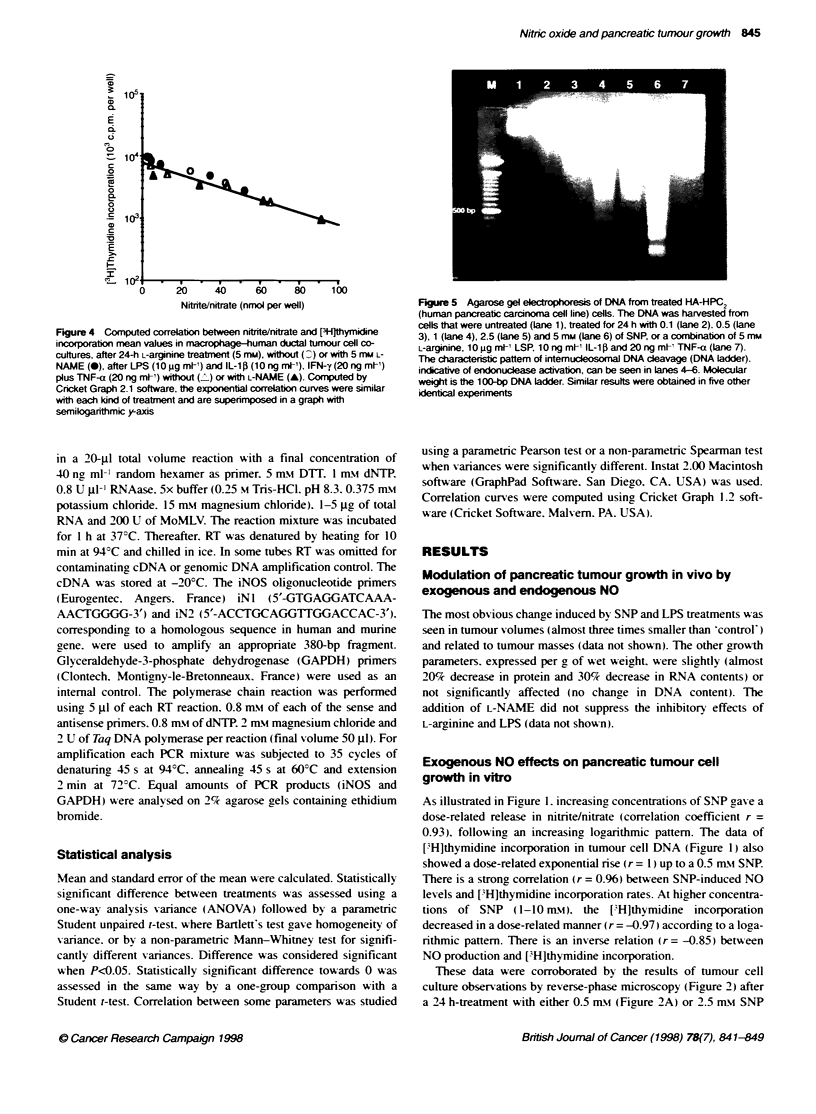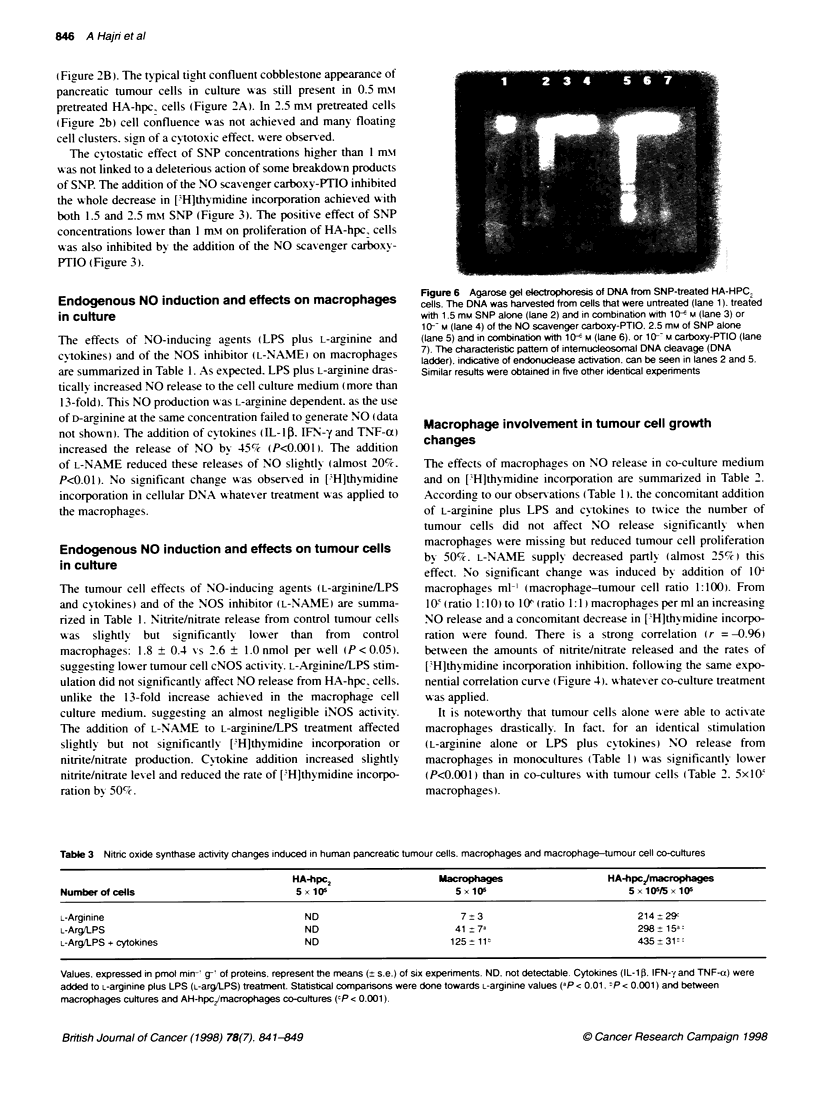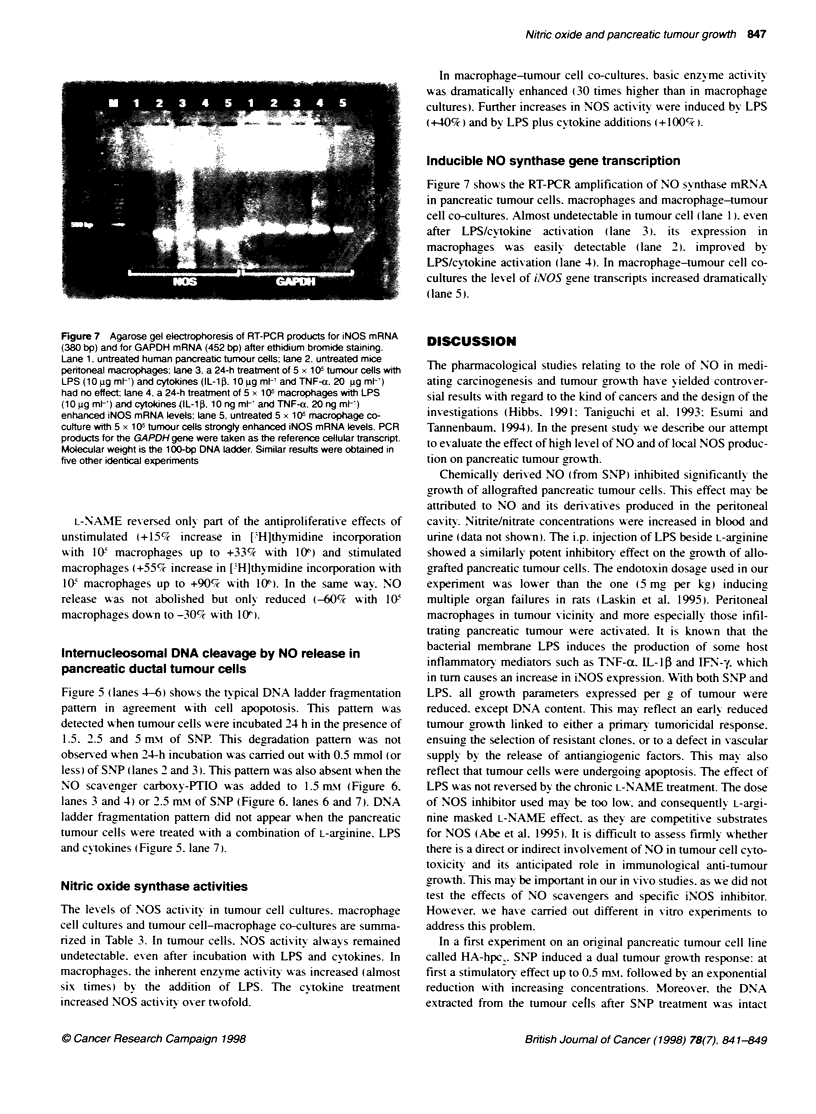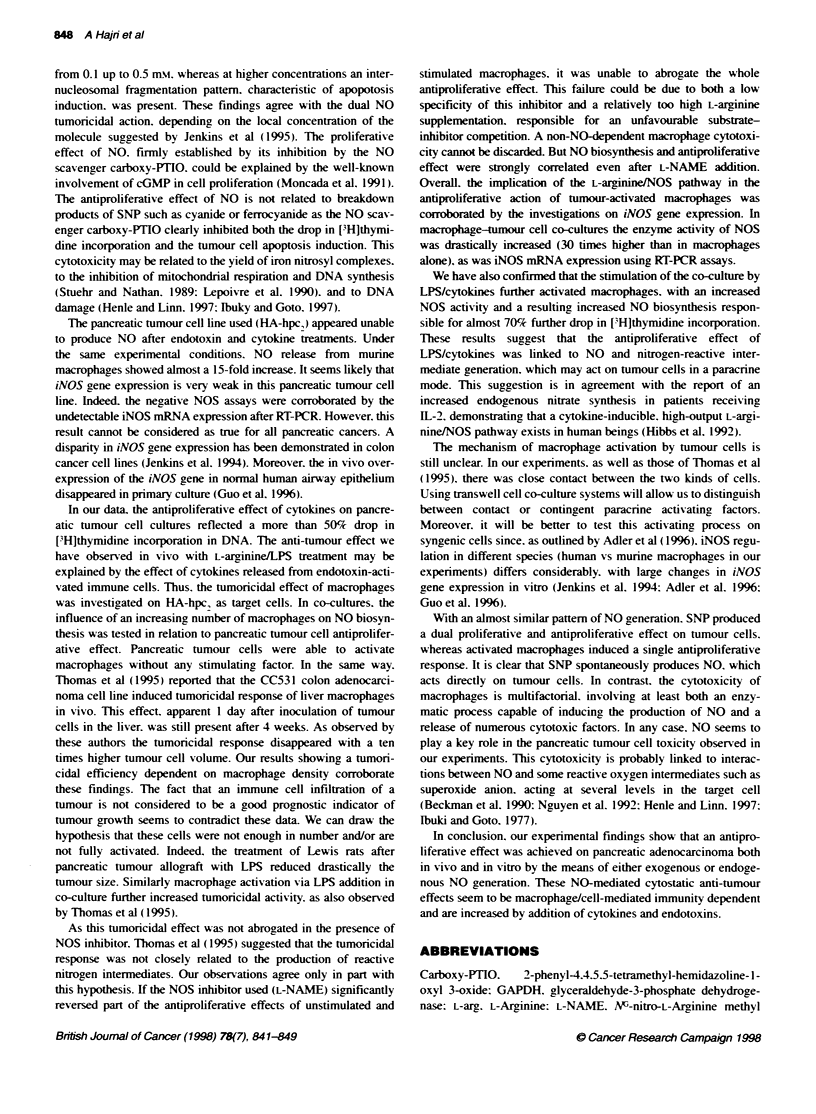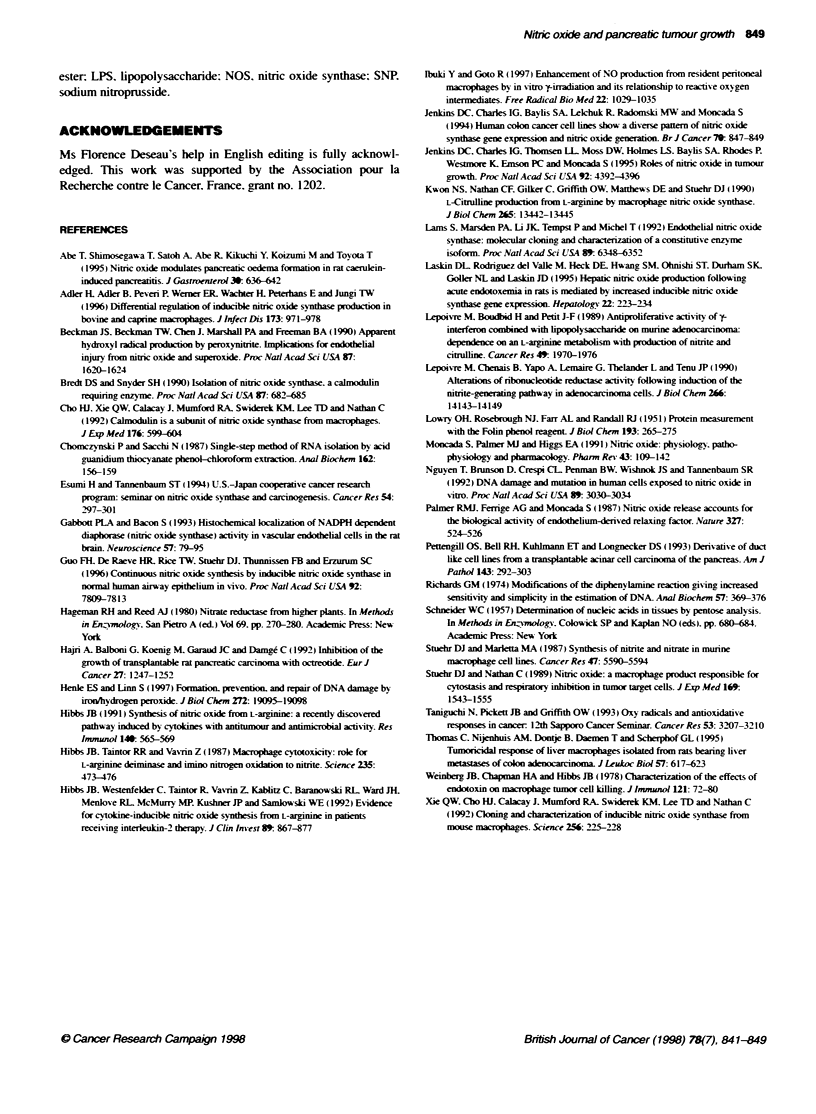# Role of nitric oxide in pancreatic tumour growth: in vivo and in vitro studies.

**DOI:** 10.1038/bjc.1998.591

**Published:** 1998-10

**Authors:** A. Hajri, E. Metzger, F. Vallat, S. Coffy, E. Flatter, S. Evrard, J. Marescaux, M. Aprahamian

**Affiliations:** Institut de Recherche contre les Cancers de l'Appareil Digestif, Hôpitaux Universitaires, Strasbourg, France.

## Abstract

**Images:**


British Journal of Cancer (1998) 78(7), 841–849
© 1998 Cancer Research Campaign

### Role of nitric oxide in pancreatic tumour growth: in vivo and in vitro studies

**A Hajri, E Metzger, F Vallat, S Coffy, E Flatter, S Evrard, J Marescaux and M Aprahamian**

Institut de Recherche contre les Cancers de l'Appareil Digestif (IRCAD), Hôpitaux Universitaires, BP 426, F-67091 Strasbourg Cedex, France

**Summary** Nitric oxide (NO), an endogenous free radical, has been implicated in a wide range of biological functions. NO is generated enzymatically from the terminal guanidinonitrogen of L-arginine by nitric oxide synthase (NOS). Despite intensive investigations, the role of NO – either as the primary product of the L-arginine/NOS pathway or provided from the NO donor sodium nitroprusside (SNP) – in carcinogenesis and tumour cell growth remains unclear and controversial. The objective of this study was to examine the growth effects of NO on a ductal pancreatic adenocarcinoma in the rat and on a human pancreatic tumour cell line (HA-hpc$_2$). In vivo, both SNP and endogenous induction of NO by endotoxins [lipopolysaccharide (LPS)] plus L-arginine significantly reduced the tumour growth. To investigate the mechanisms of NO anti-tumour growth action, the effects of either the SNP or L-arginine/NOS pathway were analysed on the HA-hpc$_2$ cell line. Nitrite/nitrate production, NOS activity and iNOS expression [assessed by reverse transcription-polymerase chain reaction (RT-PCR)] were tested and related to growth (assessed by [$^3$H]thymidine incorporation assay) and apoptosis (assessed by internucleosomal DNA cleavage). SNP exerted a dual effect on tumour cells: stimulation of the proliferation up to 1 mM and inhibition at higher concentrations. These effects were related to NO production. Both proliferative and cytostatic responses were inhibited by NO scavenger 2-phenyl-4,4,5,5-tetramethyl-hemidazoline-l-oxyl3-oxide (carboxy-PTIO). The marked apoptotic DNA fragmentation induced by SNP was also abolished by PTIO association. Unlike macrophages, the human pancreatic tumour cells did not seem to express intrinsically the L-arginine/NOS pathway. Macrophages were activated by HA-hpc$_2$ cells as well as by LPS plus cytokines [interleukin (IL)-1β plus tumour necrosis factor (TNF)-α and interferon (IFN)-γ]. In HA-hpc$_2$/macrophage co-cultures, NOS activity and inducible NOS (iNOS) transcription were stimulated, whereas an antiproliferative response was observed. These effects were related to both macrophage amount and NO production. Addition of LPS plus cytokines to co-cultures doubled iNOS activity, nitrite/nitrate production and tumoricidal effect. These data suggest the involvement of NO in pancreatic tumour growth and support the fact that generation of high levels of NO with potential production of endogenous reactive nitrogen intermediates may contribute to induction of apoptosis and tumour growth inhibition.

**Keywords:** macrophage; nitric oxide; nitric oxide synthase; pancreas; tumour growth

Nitric oxide (NO), first identified as an endothelium-derived relaxing factor (Palmer et al, 1987), emerges as a striking signal transduction molecule involved in a large variety of pathophysiological processes (Moncada et al, 1991). NO is generated as a free radical through a NO synthase-mediated oxidation (requiring additional NADPH) (Gabott and Bacon, 1993) of the terminal guanidine nitrogen of L-arginine. As demonstrated by molecular cloning (Lams et al, 1992; Xie et al, 1992), there are at least two NO synthase isoform classes: a constitutive (cNOS) or $Ca^{2+}$-calmodulin-dependent one and an inducible (iNOS) or $Ca^{2+}$-independent one, even though this class has been shown to have a calmodulin binding site (Bredt and Snyder, 1990; Moncada et al, 1991; Cho et al, 1992). Prevalent in some cell types such as endothelial (eNOS) or neuron (nNOS) cells, cNOS isoforms are activated via intracellular $Ca^{2+}$ increase or physical stimulus. Requiring calmodulin but not $Ca^{2+}$ increase for its activity, iNOS generates negligible NO under basal conditions. Inducible in macrophages, hepatocytes, endothelial or vascular smooth muscle cells, its transcription requires cytokine [interferon (IFN)-γ, tumour necrosis factor (TNF)-α and interleukin (IL)-1β] and/or endotoxin activation (Hibbs et al, 1987; Stuehr and Marletta, 1987; Lepoivre et al, 1989).

It has been long recognized that activated macrophages produce cytotoxic or at least cytostatic effects on microbial pathogens and tumour cells (Weinberg et al, 1978), via a release of both cytokines and NO (Palmer et al, 1987; Kwon et al, 1990; Hibbs, 1991). In addition, bacterial lipopolysaccharide (LPS) or cytokine iNOS activation induces macrophage NO synthesis (Stuehr and Marletta, 1987). Acting as a powerful reducing and oxidizing agent able to donate and catch electrons (Beckman et al, 1990), NO can interfere with iron–sulphur-containing enzymes ensuing iron release and inhibit both mitochondrial electron transport chain (Stuehr and Nathan, 1989) and ribonucleotide reductase activity (Lepoivre et al, 1990), a rate-limiting step in DNA synthesis. As a matter of fact, the potential consequence of producing large amounts of NO may be local cytotoxicity. As a radical, NO reacts with oxygen species and water, forming other tissue toxic N-oxides such as peroxynitrite (Beckman et al, 1990; Esumi and Tannenbaum, 1994). The role of NO and its closely related catabolic products such as $NO_2^-/NO_3^-$ in the pathogenesis of inflammation and tumour cell death or apoptosis (Nguyen et al, 1992; Esumi and Tannenbaum, 1994) can be explained this way. Excessive NOS induction, leading to too large a quantity of NO

*Received 5 August 1997*
*Revised 17 March 1998*
*Accepted 18 March 1998*

*Correspondence to:* M Aprahamian production, has also been implicated in endothelial cell damage (Beckman et al, 1990). Accumulation of all these highly reactive compounds (NO radical, nitrite, nitrate, peroxynitrite and peroxynitrate) suggests potential genotoxic effects of NO in target cells (Nguyen et al, 1992).

We have designed the present study to evaluate both the effect of pancreatic tumour cell growth inhibition by NO overproduction and the possible role of macrophage-derived NO in mediating tumoricidal activity in vivo and in vitro.

### MATERIALS AND METHODS

### Media and reagents

$N^G$-nitro-L-arginine methyl ester (L-NAME), L-arginine and D-arginine hydrochlorides, sulphanilamide, *N*-(naphthyl)ethylenediamine dihydrochloride, FAD, lipopolysaccharide (LPS), NADPH, nitrate reductase, sodium nitroprusside (SNP), sodium nitrate, sulphalinic acid, Dowex AG50 W-X8, aprotinin, leupeptin and carboxy-PTIO were purchased from Sigma-France. DMEM, RPMI, Hanks' balanced salt, fetal calf serum and antibiotics were provided from Gibco BRL (Cergy-Pontoise, France). Recombinant TNF-α, IFN-γ and IL-1β were obtained from Euromedex (Strasbourg, France). [$^3$H]thymidine (specific activity of 5 Ci mmol$^{-1}$) and L-[2,3,4,5-$^3$H] arginine (specific activity of 68 Ci mmol$^{-1}$) from Amersham (Les Ulis, France). MoMLV, reverse transcriptase and *Taq* polymerase were purchased from Appligène (Strasbourg, France) and iNOS oligonucleotide primers from Eurogentec (Angers, France).

### In vivo experiments

#### Experimental animals

Male Lewis rats (180–200 g) and male $C_3H$/HeN mice (6–10 weeks old) purchased from Charles River Laboratories (Orléans, France) were maintained according to the guidelines for laboratory animal use and care with free access to tap water and standard specific chow.

#### Tumour system

The rat ductal pancreatic carcinoma is derived from a primary transplantable acinar carcinoma that was originally chemically induced by azaserine in Lewis rats (Pettengill et al, 1993). Tumour cell preparation has been described previously (Hajri et al, 1992). Briefly, tumour tissue from the donor rat was removed, washed in ice-cold Hank's balanced salt, sliced, passed through a no. 30 stainless-steel screen and centrifuged (500 *g*). For tumour injection, the pellet was resuspended (v/v) in DMEM before cell count. Aliquots of approximately 150 µl of the final suspension of almost $5\times10^6$ cells were injected during laparotomy at a single site into the pancreas of 24 ketamin anaesthetized rats, as described elsewhere (Hajri et al, 1992). Ten days later (a time interval for tumour to attain a volume of 0.3–0.5 cm$^3$) rats were randomly allocated to four experimental groups of eight animals.

#### Experimental schedules

Animals were injected daily i.p. for 15 days with either 500 µl of sodium chloride solution (9 g l$^{-1}$), or 100 mg kg$^{-1}$ sodium nitroprusside, or 400 mg kg$^{-1}$ L-arginine monohydrochloride alone or added to 50 mg kg$^{-1}$ L-NAME. The last two groups also received a 3 mg kg$^{-1}$ endotoxin (LPS) i.p. injection at 5-day intervals. After euthanasia using an injectable agent (ketamine i.p.), tumours were removed, weighed and measured with a calliper. Pancreatic tumour contents in protein, RNA and DNA were determined according to the methods of Lowry et al (1951), Schneider (1957) and Richards (1974) respectively.

### In vitro experiments

#### Cell cultures and experimental procedures

The human pancreatic tumour cell line and the murine peritoneal macrophages were routinely cultured in DMEM or RPMI medium supplemented with 2 mM L-glutamine, 100 U ml$^{-1}$ penicillin, 100 µg ml$^{-1}$ streptomycin and 10% fetal calf serum. The cell cultures were maintained at 37°C in a humidified incubator containing 5% carbon dioxide in air.

Harvested in ice-cooled PBS plus 25 mM glucose from C3H/HeN mice treated 48 h before with 1.5 ml of 10% thioglycollate broth, macrophages were pelleted at 4°C, washed twice and counted. Then, $5\times10^5$ macrophages per ml of medium were seeded in plastic dishes (1 ml per well) and plated for initial cell cultures. After a 2-h incubation, non-adherent cells were removed by extensive PBS washing and remaining adherent macrophages were cultured in fresh medium for 24 h. Then, the medium was changed and 5 mM L-arginine monochloride and specific NO inducers (10 µg ml$^{-1}$ LPS, 10 ng ml$^{-1}$ IL-1β plus 20 ng ml$^{-1}$ IFN-γ and 20 ng ml$^{-1}$ TNF-α) were added, alone or in combination, for 24 h with or without NO inhibitor (5 mM L-NAME).

An original pancreatic tumour cell line (called HA-hpc$_2$) was used as tumour target cells. This cell line, derived from metastatic liver tumour of a human pancreatic adenocarcinoma, was established in our laboratory in tissue culture and in nude mouse (with a preserved carcinogenicity). This cell line produced ductal tumour markers such as mucine plus carbohydrate antigen 19-9 and did not express acinar cell markers such as amylase (unpublished data). In these experiments, HA-hpc$_2$ cells were cultured in a DMEM medium ($5\times10^5$ cells ml$^{-1}$) supplemented as described above for 24 h. Then, the medium was changed and specific NO donor (SNP, from 0 to 10 mM) was added for 24 h with or without carboxy-PTIO ($10^{-7}$ and $10^{-6}$ M) as NO scavenger, or 5 mM L-arginine monochloride and specific NO inducers were added for 24 h (10 µg ml$^{-1}$ LPS, with or without 10 ng ml$^{-1}$ IL-1β plus 20 ng ml$^{-1}$ IFN-γ and 20 ng ml$^{-1}$ TNF-α), with or without NO inhibitor (5 mM L-NAME).

Co-culture of macrophages with pancreatic tumour cells was also performed. At the time of macrophage monolayer medium renewal, cells were harvested, counted and an increasing amount ($10^4$, $10^5$, $5\times10^5$ and $10^6$ cells) was mixed in 24-well plates with tumour cells (initially $10^6$ cells) growing for 24 h in 1 ml of DMEM medium. These co-cultures were supplemented in 5 mM L-arginine alone or with NO inducers (10 µg ml$^{-1}$ LPS, with or without 10 ng ml$^{-1}$ IL-1β plus 20 ng ml$^{-1}$ IFN-γ and 20 ng ml$^{-1}$ TNF-α), with or without the NO inhibitor (5 mM L-NAME) and incubated in a humidified 5% carbon dioxide incubator for 24 h.

#### Assay of nitrate/nitrite

NO release was appraised by the determination of accumulating oxidation products in cell culture supernatant. Nitrite concentrations in phenol red-free medium were measured by the colorimetric Griess reaction (Hageman and Reed, 1980) after reducing nitrates to nitrites using nitrate reductase in presence of FAD (5 µM) and NADPH (50 µM). After nitrate had been reduced to

© Cancer Research Campaign 1998

**Figure 1** Effects of increasing sodium nitroprusside concentrations on human pancreatic ductal tumour cell [$^3$H]thymidine incorporation (○) and nitrite/nitrate release in cell culture medium (●) 24 h after supply. Results are the means (± s.e.) of six experiments. The dose-correlated exponential or logarithmic curves were processed by Cricket Graph 2.1 software. All changes are significantly different ($P$<0.001) from control values (no sodium nitroprusside), except for [$^3$H]thymidine incorporation achieved with 0.1 mM sodium nitroprusside nitrite, excess of NADPH, which interfered with the subsequent nitrite determination, was oxidized with L-lactic dehydrogenase and sodium pyruvate. The Griess reagent was prepared by mixing equal volumes of sulphanilamide (1% in 2.5% phosphoric acid) and *N*-(naphthyl)ethylene-diamine dihydrochloride (0.1% in 2.5% phosphoric acid). Sample aliquots of 100 µl (diluted if needed) were transferred to 96-well microassay plates and incubated for 10 min at room temperature in the dark, after addition of an equal volume of Griess reagent. Absorbance of the chromophore formed was measured at 540 nm and nitrite was quantified by using sodium nitrite as a standard. Nitrite concentration was expressed in nmol ml$^{-1}$.

### *Extraction and measurement of nitric oxide synthase activity*

Cells were washed with PBS, scraped and suspended in a lysis buffer [40 mM HEPES, pH 7.4, 32 mM sucrose, 1 mM DTT, 2 µg ml$^{-1}$ aprotinin, 10 µg ml$^{-1}$ leupeptin and 10 µg ml$^{-1}$ soybean trypsin inhibitor (SBTI)]. Cell lysis was achieved by freeze thaw: the suspension was centrifuged at 10 000 *g* for 30 min at 4°C. A Dowex-AG 50 W-X8 ($Na^+$ form) column (prepared from the $H^+$ form resin by 1 N sodium hydroxide and purified water washing until pH reached almost 6) was used to remove endogenous arginine from the cell lysate. NOS activity was then assayed by measuring the enzymatic conversion of [$^3$H]L-arginine to [$^3$H]L-citrulline (Laskin et al, 1995) in the cell extract eluates. A sample of cell extract eluate (40 µl) was incubated for 45 min at 37°C in presence of a reaction buffer (40 mM Tris/HCl, pH 8.0, 0.2 mM NADPH, 4 µM flavine adenine dinucleotide, 6 mM valine as arginase inhibitor, 0.2 mM calcium chloride, 3 mM DTT, 10 mM L-arginine and 3.5 µM L-[2,3,4,5-$^3$H]arginine. The reaction was stopped in ice and 500 µl of a water/Dowex-AG 50 W-X8 mixture (vol/vol) was added. The resin was washed twice with 1 ml of purified water, and 1 ml of supernatant was used for radioactivity determination by scintillation counting in presence of 2 ml of

**Figure 2** Reverse-phase microscopic aspects of HA-hpc$_2$ pancreatic tumour cells after a 48-h culture with 0.5 (**A**) or 2.5 mM (**B**) sodium nitroprusside 24-h treatment. The 0.5 mM sodium nitroprusside-treated culture had the typical cobblestone appearance of pancreatic duct cells in culture. The 2.5 mM sodium nitroprusside treatment produced obvious changes in cell number and aspect with some pyknotic figures (actual magnification × 10)

**Figure 3** Effects of the NO scavenger carboxy-PTIO on [$^3$H]thymidine incorporation in DNA of HA-hpc$^2$ tumour cells treated with increasing sodium nitroprusside concentrations. Sodium nitroprusside alone, ■; $10^{-6}$ M carboxy-PTIO, □; $10^{-7}$ M carboxy-PTIO, ▥. Results are mean (± s.e.) of six experiments. Statistical comparisons were done towards untreated cells. ***$P$<0.001

Pico-Fluor liquid. Nitric oxide synthase activity was expressed in pmol $min^{-1}$ $g^{-1}$ of protein.

### [$^3$H]thymidine incorporation assay

Tumour cell proliferation after each kind of treatment was assessed after 1 day of culture by determining the [$^3$H]thymidine incorporation into DNA. Briefly, 0.5 µCi $ml^{-1}$ of [*methyl*-$^3$H]thymidine was added and cells were incubated for 24 h. After PBS washing, cells were incubated for 20 min in ice-cold 10% TCA, rinsed and lysed by adding 750 µl per well of sodium hydroxide 0.4 N for 30 min at 37°C. The amount of radioactivity incorporated in DNA per well was determined by liquid scintillation spectrometry of the cell lysate. [$^3$H]Thymidine incorporation was expressed in c.p.m. per well.

### Electrophoretic detection of internucleosomal DNA cleavage

After each specific treatment, tumour cells were pelleted at 4°C, resuspended in a lysis buffer (0.2 M Tris/HCl pH 8.0, 20 mM EDTA, 100 µg $ml^{-1}$ proteinase K and 10 µg $ml^{-1}$ SDS) and incubated for 4 h at 37°C. After RNAase treatment (100 µg $ml^{-1}$) for 1 h at 37°C, DNA was extracted with a phenol–chloroform mixture (vol/vol) and precipitated with sodium acetate 3 M (1/10 vol) and ice-cold ethanol (2.5 vol) for 1 h at room temperature. DNA samples and a 100-bp DNA ladder (Promega, Charbonnières, France) were mixed with loading buffer (30% glycerol, 0.1% bromophenol blue) and loaded onto 1.8% agarose gels and electrophoresed for approximately 1 h at 50 V. The gels were stained with ethidium bromide, and the DNA bands were visualized under 312-nm light.

### RT-PCR of inducible NO synthase mRNA

Single-strand cDNA was reverse transcribed from total RNA, extracted by the guanidinium isothiocyanate method (Chomczynski and Sacchi, 1987), using Moloney murine leukaemia virus (MoMLV) reverse transcriptase (RT) and $(dT)_{12-18}$ primer or random hexamers. Reverse transcription was carried out

**Table 1** Endogenous nitric oxide induction and effects on macrophage and pancreatic tumour cell growth

| | Nitrite/nitrate (nmol per well) | | [$^3$H]Thymidine (c.p.m. per well) | |
|---|---|---|---|---|
| | No L-NAME | + L-NAME | No L-NAME | + L-NAME |
| Murine macrophages | | | | |
| L-Arginine | 2.6 ± 1.0 | – | 106 ± 10 | – |
| L-Arg/LPS | 30.2 ± 1.3[a] | 23.1 ± 0.9[a,c] | 102 ± 12 | 91 ± 10 |
| L-Arg/LPS + cytokines | 44.2 ± 1.4[a] | 37.0 ± 1.5[a,c] | 108 ± 16 | 113 ± 17 |
| Human pancreatic tumour cells | | | | |
| L-Arginine | 1.8 ± 0.4 | – | 4838 ± 366 | – |
| L-Arg/LPS | 2.2 ± 0.4 | 1.4 ± 0.2 | 3400 ± 241[b] | 3821 ± 153[c] |
| L-Arg/LPS + cytokines | 3.1 ± 0.4 | – | 1753 ± 125[a] | – |

Values represent the means (± s.e.) of six experiments. Each well contains 5 × $10^5$ cells in 1 ml of medium. Cytokines (IL-1β, IFN-γ and TNF-α) were added to L-arg/LPS treatment (L-arginine and LPS stimulation). The NOS inhibitor (L-NAME) was tested on L-arg/LPS experiments. Statistical comparisons were done towards L-arginine control values ($^aP < 0.001$, $^bP < 0.01$) and, when L-NAME treatment was applied, towards the corresponding L-arg/LPS group ($^cP < 0.01$).

**Table 2** Effects of increasing concentration of macrophages on human pancreatic tumour cell growth

| $10^6$ tumour cells in addition to | Nitrite/nitrate (nmol per well) | | [$^3$H]Thymidine (c.p.m. per well) | |
|---|---|---|---|---|
| | No L-NAME | + L-NAME | No L-NAME | + L-NAME |
| No macrophage | | | | |
| + L-Arginine | 3.0 ± 1.0 | 2.8 ± 0.8 | 10295 ± 304 | 10561 ± 333 |
| + LPS + cytokines | 5.6 ± 1.1 | 4.1 ± 1.9 | 4939 ± 3.8[c] | 6945 ± 297[c] |
| $10^4$ macrophages | | | | |
| + L-Arginine | 3.1 ± 0.9 | 2.1 ± 0.6 | 10627 ± 293 | 10968 ± 368 |
| + LPS + cytokines | 5.5 ± 1.4 | 4.1 ± 2.1 | 5298 ± 296[c] | 7708 ± 517[c] |
| $10^5$ macrophages | | | | |
| + L-Arginine | 8.9 ± 1.3 | 4.1 ± 0.7[b] | 8252 ± 283 | 9505 ± 511 |
| + LPS + cytokines | 29.2 ± 5.7[b] | 12.6 ± 1.7 | 3637 ± 222[c] | 5655 ± 462[c] |
| 5 × $10^5$ macrophages | | | | |
| + L-Arginine | 34.1 ± 2.2 | 24.1 ± 1.6[b] | 5393 ± 180 | 6329 ± 484 |
| + LPS + cytokines | 61.5 ± 2.8[c] | 43.3 ± 3.9 | 1962 ± 192[c] | 3555 ± 427[b] |
| $10^6$ macrophages | | | | |
| + L-Arginine | 51.9 ± 3.3 | 37.1 ± 2.8[b] | 2785 ± 225 | 3887 ± 443 |
| + LPS + cytokines | 91.2 ± 5.5[c] | 65.2 ± 4.2[a] | 807 ± 106[c] | 1738 ± 357[a] |

Values represent the means (± s.e.) of six experiments. Co-cultures supplemented in L-arginine alone served as controls. Both LPS and cytokines (IL-1β, IFN-γ and TNF-α) were added to co-culture medium. All the experiments were performed with and without the NOS inhibitor (L-NAME). For each macrophage concentration, statistical comparisons were done towards the corresponding L-arginine-no L-NAME control values ($^aP < 0.05$, $^bP < 0.01$, $^cP < 0.001$).

© Cancer Research Campaign 1998

**Figure 4** Computed correlation between nitrite/nitrate and [$^3$H]thymidine incorporation mean values in macrophage–human ductal tumour cell co-cultures, after 24-h L-arginine treatment (5 mM), without (○) or with 5 mM L-NAME (●), after LPS (10 μg ml$^{-1}$) and IL-1β (10 ng ml$^{-1}$), IFN-γ (20 ng ml$^{-1}$) plus TNF-α (20 ng ml$^{-1}$) without (△) or with L-NAME (▲). Computed by Cricket Graph 2.1 software, the exponential correlation curves were similar with each kind of treatment and are superimposed in a graph with semilogarithmic y-axis

**Figure 5** Agarose gel electrophoresis of DNA from treated HA-HPC$_2$ (human pancreatic carcinoma cell line) cells. The DNA was harvested from cells that were untreated (lane 1), treated for 24 h with 0.1 (lane 2), 0.5 (lane 3), 1 (lane 4), 2.5 (lane 5) and 5 mM (lane 6) of SNP, or a combination of 5 mM L-arginine, 10 μg ml$^{-1}$ LSP, 10 ng ml$^{-1}$ IL-1β and 20 ng ml$^{-1}$ TNF-α (lane 7). The characteristic pattern of internucleosomal DNA cleavage (DNA ladder), indicative of endonuclease activation, can be seen in lanes 4–6. Molecular weight is the 100-bp DNA ladder. Similar results were obtained in five other identical experiments in a 20-μl total volume reaction with a final concentration of 40 ng ml$^{-1}$ random hexamer as primer, 5 mM DTT, 1 mM dNTP, 0.8 U μl$^{-1}$ RNAase, 5× buffer (0.25 M Tris-HCl, pH 8.3, 0.375 mM potassium chloride, 15 mM magnesium chloride), 1–5 μg of total RNA and 200 U of MoMLV. The reaction mixture was incubated for 1 h at 37°C. Thereafter, RT was denatured by heating for 10 min at 94°C and chilled in ice. In some tubes RT was omitted for contaminating cDNA or genomic DNA amplification control. The cDNA was stored at –20°C. The iNOS oligonucleotide primers (Eurogentec, Angers, France) iN1 (5′-GTGAGGATCAAA-AACTGGGG-3′) and iN2 (5′-ACCTGCAGGTTGGACCAC-3′), corresponding to a homologous sequence in human and murine gene, were used to amplify an appropriate 380-bp fragment. Glyceraldehyde-3-phosphate dehydrogenase (GAPDH) primers (Clontech, Montigny-le-Bretonneaux, France) were used as an internal control. The polymerase chain reaction was performed using 5 μl of each RT reaction, 0.8 mM of each of the sense and antisense primers, 0.8 mM of dNTP, 2 mM magnesium chloride and 2 U of *Taq* DNA polymerase per reaction (final volume 50 μl). For amplification each PCR mixture was subjected to 35 cycles of denaturing 45 s at 94°C, annealing 45 s at 60°C and extension 2 min at 72°C. Equal amounts of PCR products (iNOS and GAPDH) were analysed on 2% agarose gels containing ethidium bromide.

### Statistical analysis

Mean and standard error of the mean were calculated. Statistically significant difference between treatments was assessed using a one-way analysis variance (ANOVA) followed by a parametric Student unpaired *t*-test, where Bartlett's test gave homogeneity of variance, or by a non-parametric Mann–Whitney test for significantly different variances. Difference was considered significant when $P<0.05$. Statistically significant difference towards 0 was assessed in the same way by a one-group comparison with a Student *t*-test. Correlation between some parameters was studied using a parametric Pearson test or a non-parametric Spearman test when variances were significantly different. Instat 2.00 Macintosh software (GraphPad Software, San Diego, CA, USA) was used. Correlation curves were computed using Cricket Graph 1.2 software (Cricket Software, Malvern, PA, USA).

### RESULTS

### Modulation of pancreatic tumour growth in vivo by exogenous and endogenous NO

The most obvious change induced by SNP and LPS treatments was seen in tumour volumes (almost three times smaller than 'control') and related to tumour masses (data not shown). The other growth parameters, expressed per g of wet weight, were slightly (almost 20% decrease in protein and 30% decrease in RNA contents) or not significantly affected (no change in DNA content). The addition of L-NAME did not suppress the inhibitory effects of L-arginine and LPS (data not shown).

### Exogenous NO effects on pancreatic tumour cell growth in vitro

As illustrated in Figure 1, increasing concentrations of SNP gave a dose-related release in nitrite/nitrate (correlation coefficient $r$ = 0.93), following an increasing logarithmic pattern. The data of [$^3$H]thymidine incorporation in tumour cell DNA (Figure 1) also showed a dose-related exponential rise ($r$ = 1) up to a 0.5 mM SNP. There is a strong correlation ($r$ = 0.96) between SNP-induced NO levels and [$^3$H]thymidine incorporation rates. At higher concentrations of SNP (1–10 mM), the [$^3$H]thymidine incorporation decreased in a dose-related manner ($r$ = –0.97) according to a logarithmic pattern. There is an inverse relation ($r$ = –0.85) between NO production and [$^3$H]thymidine incorporation.

These data were corroborated by the results of tumour cell culture observations by reverse-phase microscopy (Figure 2) after a 24 h-treatment with either 0.5 mM (Figure 2A) or 2.5 mM SNP

© Cancer Research Campaign 1998

(Figure 2B). The typical tight confluent cobblestone appearance of pancreatic tumour cells in culture was still present in 0.5 mM pretreated HA-hpc$_2$ cells (Figure 2A). In 2.5 mM pretreated cells (Figure 2b) cell confluence was not achieved and many floating cell clusters, sign of a cytotoxic effect, were observed.

The cytostatic effect of SNP concentrations higher than 1 mM was not linked to a deleterious action of some breakdown products of SNP. The addition of the NO scavenger carboxy-PTIO inhibited the whole decrease in [$^3$H]thymidine incorporation achieved with both 1.5 and 2.5 mM SNP (Figure 3). The positive effect of SNP concentrations lower than 1 mM on proliferation of HA-hpc$_2$ cells was also inhibited by the addition of the NO scavenger carboxy-PTIO (Figure 3).

### Endogenous NO induction and effects on macrophages in culture

The effects of NO-inducing agents (LPS plus L-arginine and cytokines) and of the NOS inhibitor (L-NAME) on macrophages are summarized in Table 1. As expected, LPS plus L-arginine drastically increased NO release to the cell culture medium (more than 13-fold). This NO production was L-arginine dependent, as the use of D-arginine at the same concentration failed to generate NO (data not shown). The addition of cytokines (IL-1β, IFN-γ and TNF-α) increased the release of NO by 45% ($P$<0.001). The addition of L-NAME reduced these releases of NO slightly (almost 20%, $P$<0.01). No significant change was observed in [$^3$H]thymidine incorporation in cellular DNA whatever treatment was applied to the macrophages.

### Endogenous NO induction and effects on tumour cells in culture

The tumour cell effects of NO-inducing agents (L-arginine/LPS and cytokines) and of the NOS inhibitor (L-NAME) are summarized in Table 1. Nitrite/nitrate release from control tumour cells was slightly but significantly lower than from control macrophages: 1.8 ± 0.4 vs 2.6 ± 1.0 nmol per well ($P$ < 0.05), suggesting lower tumour cell cNOS activity. L-Arginine/LPS stimulation did not significantly affect NO release from HA-hpc$_2$ cells, unlike the 13-fold increase achieved in the macrophage cell culture medium, suggesting an almost negligible iNOS activity. The addition of L-NAME to L-arginine/LPS treatment affected slightly but not significantly [$^3$H]thymidine incorporation or nitrite/nitrate production. Cytokine addition increased slightly nitrite/nitrate level and reduced the rate of [$^3$H]thymidine incorporation by 50%.

**Figure 6** Agarose gel electrophoresis of DNA from SNP-treated HA-HPC$_2$ cells. The DNA was harvested from cells that were untreated (lane 1), treated with 1.5 mM SNP alone (lane 2) and in combination with $10^{-6}$ M (lane 3) or $10^{-7}$ M (lane 4) of the NO scavenger carboxy-PTIO. 2.5 mM of SNP alone (lane 5) and in combination with $10^{-6}$ M (lane 6), or $10^{-7}$ M carboxy-PTIO (lane 7). The characteristic pattern of internucleosomal DNA cleavage (DNA ladder), indicative of endonuclease activation, can be seen in lanes 2 and 5. Similar results were obtained in five other identical experiments

### Macrophage involvement in tumour cell growth changes

The effects of macrophages on NO release in co-culture medium and on [$^3$H]thymidine incorporation are summarized in Table 2. According to our observations (Table 1), the concomitant addition of L-arginine plus LPS and cytokines to twice the number of tumour cells did not affect NO release significantly when macrophages were missing but reduced tumour cell proliferation by 50%. L-NAME supply decreased partly (almost 25%) this effect. No significant change was induced by addition of $10^4$ macrophages ml$^{-1}$ (macrophage–tumour cell ratio 1:100). From $10^5$ (ratio 1:10) to $10^6$ (ratio 1:1) macrophages per ml an increasing NO release and a concomitant decrease in [$^3$H]thymidine incorporation were found. There is a strong correlation ($r$ = –0.96) between the amounts of nitrite/nitrate released and the rates of [$^3$H]thymidine incorporation inhibition, following the same exponential correlation curve (Figure 4), whatever co-culture treatment was applied.

It is noteworthy that tumour cells alone were able to activate macrophages drastically. In fact, for an identical stimulation (L-arginine alone or LPS plus cytokines) NO release from macrophages in monocultures (Table 1) was significantly lower ($P$<0.001) than in co-cultures with tumour cells (Table 2, 5×10$^5$ macrophages).

**Table 3** Nitric oxide synthase activity changes induced in human pancreatic tumour cells, macrophages and macrophage–tumour cell co-cultures

| Number of cells | HA-hpc$_2$ 5 × 10$^5$ | Macrophages 5 × 10$^5$ | HA-hpc$_2$/macrophages 5 × 10$^5$/5 × 10$^5$ |
|---|---|---|---|
| L-Arginine | ND | 7 ± 3 | 214 ± 29$^c$ |
| L-Arg/LPS | ND | 41 ± 7$^a$ | 298 ± 15$^{a,c}$ |
| L-Arg/LPS + cytokines | ND | 125 ± 11$^b$ | 435 ± 31$^{b,c}$ |

Values, expressed in pmol min$^{-1}$ g$^{-1}$ of proteins, represent the means (± s.e.) of six experiments. ND, not detectable. Cytokines (IL-1β, IFN-γ and TNF-α) were added to L-arginine plus LPS (L-arg/LPS) treatment. Statistical comparisons were done towards L-arginine values ($^a$$P$ < 0.01, $^b$$P$ < 0.001) and between macrophages cultures and AH-hpc$_2$/macrophages co-cultures ($^c$$P$ < 0.001).

 © Cancer Research Campaign 1998

**Figure 7** Agarose gel electrophoresis of RT-PCR products for iNOS mRNA (380 bp) and for GAPDH mRNA (452 bp) after ethidium bromide staining. Lane 1, untreated human pancreatic tumour cells; lane 2, untreated mice peritoneal macrophages; lane 3, a 24-h treatment of 5 × 10$^5$ tumour cells with LPS (10 μg ml$^{-1}$) and cytokines (IL-1β, 10 μg ml$^{-1}$ and TNF-α, 20 μg ml$^{-1}$) had no effect; lane 4, a 24-h treatment of 5 × 10$^5$ macrophages with LPS (10 μg ml$^{-1}$) and cytokines (IL-1β, 10 ng ml$^{-1}$ and TNF-α, 20 ng ml$^{-1}$) enhanced iNOS mRNA levels; lane 5, untreated 5 × 10$^5$ macrophage co-culture with 5 × 10$^5$ tumour cells strongly enhanced iNOS mRNA levels. PCR products for the *GAPDH* gene were taken as the reference cellular transcript. Molecular weight is the 100-bp DNA ladder. Similar results were obtained in five other identical experiments

L-NAME reversed only part of the antiproliferative effects of unstimulated (+15% increase in [$^3$H]thymidine incorporation with 10$^5$ macrophages up to +33% with 10$^6$) and stimulated macrophages (+55% increase in [$^3$H]thymidine incorporation with 10$^5$ macrophages up to +90% with 10$^6$). In the same way, NO release was not abolished but only reduced (–60% with 10$^5$ macrophages down to –30% with 10$^6$).

### Internucleosomal DNA cleavage by NO release in pancreatic ductal tumour cells

Figure 5 (lanes 4–6) shows the typical DNA ladder fragmentation pattern in agreement with cell apopotosis. This pattern was detected when tumour cells were incubated 24 h in the presence of 1.5, 2.5 and 5 mM of SNP. This degradation pattern was not observed when 24-h incubation was carried out with 0.5 mmol (or less) of SNP (lanes 2 and 3). This pattern was also absent when the NO scavenger carboxy-PTIO was added to 1.5 mM (Figure 6, lanes 3 and 4) or 2.5 mM of SNP (Figure 6, lanes 6 and 7). DNA ladder fragmentation pattern did not appear when the pancreatic tumour cells were treated with a combination of L-arginine, LPS and cytokines (Figure 5, lane 7).

### Nitric oxide synthase activities

The levels of NOS activity in tumour cell cultures, macrophage cell cultures and tumour cell–macrophage co-cultures are summarized in Table 3. In tumour cells, NOS activity always remained undetectable, even after incubation with LPS and cytokines. In macrophages, the inherent enzyme activity was increased (almost six times) by the addition of LPS. The cytokine treatment increased NOS activity over twofold.

In macrophage–tumour cell co-cultures, basic enzyme activity was dramatically enhanced (30 times higher than in macrophage cultures). Further increases in NOS activity were induced by LPS (+40%) and by LPS plus cytokine additions (+100%).

### Inducible NO synthase gene transcription

Figure 7 shows the RT-PCR amplification of NO synthase mRNA in pancreatic tumour cells, macrophages and macrophage–tumour cell co-cultures. Almost undetectable in tumour cell (lane 1), even after LPS/cytokine activation (lane 3), its expression in macrophages was easily detectable (lane 2), improved by LPS/cytokine activation (lane 4). In macrophage–tumour cell co-cultures the level of *iNOS* gene transcripts increased dramatically (lane 5).

### DISCUSSION

The pharmacological studies relating to the role of NO in mediating carcinogenesis and tumour growth have yielded controversial results with regard to the kind of cancers and the design of the investigations (Hibbs, 1991; Taniguchi et al, 1993; Esumi and Tannenbaum, 1994). In the present study we describe our attempt to evaluate the effect of high level of NO and of local NOS production on pancreatic tumour growth.

Chemically derived NO (from SNP) inhibited significantly the growth of allografted pancreatic tumour cells. This effect may be attributed to NO and its derivatives produced in the peritoneal cavity. Nitrite/nitrate concentrations were increased in blood and urine (data not shown). The i.p. injection of LPS beside L-arginine showed a similarly potent inhibitory effect on the growth of allografted pancreatic tumour cells. The endotoxin dosage used in our experiment was lower than the one (5 mg per kg) inducing multiple organ failures in rats (Laskin et al, 1995). Peritoneal macrophages in tumour vicinity and more especially those infiltrating pancreatic tumour were activated. It is known that the bacterial membrane LPS induces the production of some host inflammatory mediators such as TNF-α, IL-1β and IFN-γ, which in turn causes an increase in iNOS expression. With both SNP and LPS, all growth parameters expressed per g of tumour were reduced, except DNA content. This may reflect an early reduced tumour growth linked to either a primary tumoricidal response, ensuing the selection of resistant clones, or to a defect in vascular supply by the release of antiangiogenic factors. This may also reflect that tumour cells were undergoing apoptosis. The effect of LPS was not reversed by the chronic L-NAME treatment. The dose of NOS inhibitor used may be too low, and consequently L-arginine masked L-NAME effect, as they are competitive substrates for NOS (Abe et al, 1995). It is difficult to assess firmly whether there is a direct or indirect involvement of NO in tumour cell cytotoxicity and its anticipated role in immunological anti-tumour growth. This may be important in our in vivo studies, as we did not test the effects of NO scavengers and specific iNOS inhibitor. However, we have carried out different in vitro experiments to address this problem.

In a first experiment on an original pancreatic tumour cell line called HA-hpc$_2$, SNP induced a dual tumour growth response: at first a stimulatory effect up to 0.5 mM, followed by an exponential reduction with increasing concentrations. Moreover, the DNA extracted from the tumour cells after SNP treatment was intact from 0.1 up to 0.5 mM, whereas at higher concentrations an internucleosomal fragmentation pattern, characteristic of apoptosis induction, was present. These findings agree with the dual NO tumoricidal action, depending on the local concentration of the molecule suggested by Jenkins et al (1995). The proliferative effect of NO, firmly established by its inhibition by the NO scavenger carboxy-PTIO, could be explained by the well-known involvement of cGMP in cell proliferation (Moncada et al, 1991). The antiproliferative effect of NO is not related to breakdown products of SNP such as cyanide or ferrocyanide as the NO scavenger carboxy-PTIO clearly inhibited both the drop in [$^3$H]thymidine incorporation and the tumour cell apoptosis induction. This cytotoxicity may be related to the yield of iron nitrosyl complexes, to the inhibition of mitochondrial respiration and DNA synthesis (Stuehr and Nathan, 1989; Lepoivre et al, 1990), and to DNA damage (Henle and Linn, 1997; Ibuky and Goto, 1997).

The pancreatic tumour cell line used (HA-hpc$_2$) appeared unable to produce NO after endotoxin and cytokine treatments. Under the same experimental conditions, NO release from murine macrophages showed almost a 15-fold increase. It seems likely that *iNOS* gene expression is very weak in this pancreatic tumour cell line. Indeed, the negative NOS assays were corroborated by the undetectable iNOS mRNA expression after RT-PCR. However, this result cannot be considered as true for all pancreatic cancers. A disparity in *iNOS* gene expression has been demonstrated in colon cancer cell lines (Jenkins et al, 1994). Moreover, the in vivo overexpression of the *iNOS* gene in normal human airway epithelium disappeared in primary culture (Guo et al, 1996).

In our data, the antiproliferative effect of cytokines on pancreatic tumour cell cultures reflected a more than 50% drop in [$^3$H]thymidine incorporation in DNA. The anti-tumour effect we have observed in vivo with L-arginine/LPS treatment may be explained by the effect of cytokines released from endotoxin-activated immune cells. Thus, the tumoricidal effect of macrophages was investigated on HA-hpc$_2$ as target cells. In co-cultures, the influence of an increasing number of macrophages on NO biosynthesis was tested in relation to pancreatic tumour cell antiproliferative effect. Pancreatic tumour cells were able to activate macrophages without any stimulating factor. In the same way, Thomas et al (1995) reported that the CC531 colon adenocarcinoma cell line induced tumoricidal response of liver macrophages in vivo. This effect, apparent 1 day after inoculation of tumour cells in the liver, was still present after 4 weeks. As observed by these authors the tumoricidal response disappeared with a ten times higher tumour cell volume. Our results showing a tumoricidal efficiency dependent on macrophage density corroborate these findings. The fact that an immune cell infiltration of a tumour is not considered to be a good prognostic indicator of tumour growth seems to contradict these data. We can draw the hypothesis that these cells were not enough in number and/or are not fully activated. Indeed, the treatment of Lewis rats after pancreatic tumour allograft with LPS reduced drastically the tumour size. Similarly macrophage activation via LPS addition in co-culture further increased tumoricidal activity, as also observed by Thomas et al (1995).

As this tumoricidal effect was not abrogated in the presence of NOS inhibitor, Thomas et al (1995) suggested that the tumoricidal response was not closely related to the production of reactive nitrogen intermediates. Our observations agree only in part with this hypothesis. If the NOS inhibitor used (L-NAME) significantly reversed part of the antiproliferative effects of unstimulated and stimulated macrophages, it was unable to abrogate the whole antiproliferative effect. This failure could be due to both a low specificity of this inhibitor and a relatively too high L-arginine supplementation, responsible for an unfavourable substrate–inhibitor competition. A non-NO-dependent macrophage cytotoxicity cannot be discarded. But NO biosynthesis and antiproliferative effect were strongly correlated even after L-NAME addition. Overall, the implication of the L-arginine/NOS pathway in the antiproliferative action of tumour-activated macrophages was corroborated by the investigations on *iNOS* gene expression. In macrophage–tumour cell co-cultures the enzyme activity of NOS was drastically increased (30 times higher than in macrophages alone), as was iNOS mRNA expression using RT-PCR assays.

We have also confirmed that the stimulation of the co-culture by LPS/cytokines further activated macrophages, with an increased NOS activity and a resulting increased NO biosynthesis responsible for almost 70% further drop in [$^3$H]thymidine incorporation. These results suggest that the antiproliferative effect of LPS/cytokines was linked to NO and nitrogen-reactive intermediate generation, which may act on tumour cells in a paracrine mode. This suggestion is in agreement with the report of an increased endogenous nitrate synthesis in patients receiving IL-2, demonstrating that a cytokine-inducible, high-output L-arginine/NOS pathway exists in human beings (Hibbs et al, 1992).

The mechanism of macrophage activation by tumour cells is still unclear. In our experiments, as well as those of Thomas et al (1995), there was close contact between the two kinds of cells. Using transwell cell co-culture systems will allow us to distinguish between contact or contingent paracrine activating factors. Moreover, it will be better to test this activating process on syngenic cells since, as outlined by Adler et al (1996), iNOS regulation in different species (human vs murine macrophages in our experiments) differs considerably, with large changes in *iNOS* gene expression in vitro (Jenkins et al, 1994; Adler et al, 1996; Guo et al, 1996).

With an almost similar pattern of NO generation, SNP produced a dual proliferative and antiproliferative effect on tumour cells, whereas activated macrophages induced a single antiproliferative response. It is clear that SNP spontaneously produces NO, which acts directly on tumour cells. In contrast, the cytotoxicity of macrophages is multifactorial, involving at least both an enzymatic process capable of inducing the production of NO and a release of numerous cytotoxic factors. In any case, NO seems to play a key role in the pancreatic tumour cell toxicity observed in our experiments. This cytotoxicity is probably linked to interactions between NO and some reactive oxygen intermediates such as superoxide anion, acting at several levels in the target cell (Beckman et al, 1990; Nguyen et al, 1992; Henle and Linn, 1997; Ibuki and Goto, 1977).

In conclusion, our experimental findings show that an antiproliferative effect was achieved on pancreatic adenocarcinoma both in vivo and in vitro by the means of either exogenous or endogenous NO generation. These NO-mediated cytostatic anti-tumour effects seem to be macrophage/cell-mediated immunity dependent and are increased by addition of cytokines and endotoxins.

### ABBREVIATIONS

Carboxy-PTIO, 2-phenyl-4,4,5,5-tetramethyl-hemidazoline-1-oxyl 3-oxide; GAPDH, glyceraldehyde-3-phosphate dehydrogenase; L-arg, L-Arginine; L-NAME, $N^G$-nitro-L-Arginine methyl

© Cancer Research Campaign 1998

ester; LPS, lipopolysaccharide; NOS, nitric oxide synthase; SNP, sodium nitroprusside.

### ACKNOWLEDGEMENTS

Ms Florence Deseau's help in English editing is fully acknowledged. This work was supported by the Association pour la Recherche contre le Cancer, France, grant no. 1202.

### REFERENCES

Abe T, Shimosegawa T, Satoh A, Abe R, Kikuchi Y, Koizumi M and Toyota T (1995) Nitric oxide modulates pancreatic oedema formation in rat caerulein-induced pancreatitis. *J Gastroenterol* **30**: 636–642

Adler H, Adler B, Peveri P, Werner ER, Wachter H, Peterhans E and Jungi TW (1996) Differential regulation of inducible nitric oxide synthase production in bovine and caprine macrophages. *J Infect Dis* **173**: 971–978

Beckman JS, Beckman TW, Chen J, Marshall PA and Freeman BA (1990) Apparent hydroxyl radical production by peroxynitrite. Implications for endothelial injury from nitric oxide and superoxide. *Proc Natl Acad Sci USA* **87**: 1620–1624

Bredt DS and Snyder SH (1990) Isolation of nitric oxide synthase, a calmodulin requiring enzyme. *Proc Natl Acad Sci USA* **87**: 682–685

Cho HJ, Xie QW, Calacay J, Mumford RA, Swiderek KM, Lee TD and Nathan C (1992) Calmodulin is a subunit of nitric oxide synthase from macrophages. *J Exp Med* **176**: 599–604

Chomczynski P and Sacchi N (1987) Single-step method of RNA isolation by acid guanidium thiocyanate phenol–chloroform extraction. *Anal Biochem* **162**: 156–159

Esumi H and Tannenbaum ST (1994) U.S.–Japan cooperative cancer research program: seminar on nitric oxide synthase and carcinogenesis. *Cancer Res* **54**: 297–301

Gabbott PLA and Bacon S (1993) Histochemical localization of NADPH dependent diaphorase (nitric oxide synthase) activity in vascular endothelial cells in the rat brain. *Neuroscience* **57**: 79–95

Guo FH, De Raeve HR, Rice TW, Stuehr DJ, Thunnissen FB and Erzurum SC (1996) Continuous nitric oxide synthesis by inducible nitric oxide synthase in normal human airway epithelium in vivo. *Proc Natl Acad Sci USA* **92**: 7809–7813

Hageman RH and Reed AJ (1980) Nitrate reductase from higher plants. In *Methods in Enzymology*, San Pietro A (ed.) Vol 69, pp. 270–280. Academic Press: New York

Hajri A, Balboni G, Koenig M, Garaud JC and Damgé C (1992) Inhibition of the growth of transplantable rat pancreatic carcinoma with octreotide. *Eur J Cancer* **27**: 1247–1252

Henle ES and Linn S (1997) Formation, prevention, and repair of DNA damage by iron/hydrogen peroxide. *J Biol Chem* **272**: 19095–19098

Hibbs JB (1991) Synthesis of nitric oxide from L-arginine: a recently discovered pathway induced by cytokines with antitumour and antimicrobial activity. *Res Immunol* **140**: 565–569

Hibbs JB, Taintor RR and Vavrin Z (1987) Macrophage cytotoxicity: role for L-arginine deiminase and imino nitrogen oxidation to nitrite. *Science* **235**: 473–476

Hibbs JB, Westenfelder C, Taintor R, Vavrin Z, Kablitz C, Baranowski RL, Ward JH, Menlove RL, McMurry MP, Kushner JP and Samlowski WE (1992) Evidence for cytokine-inducible nitric oxide synthesis from L-arginine in patients receiving interleukin-2 therapy. *J Clin Invest* **89**: 867–877

Ibuki Y and Goto R (1997) Enhancement of NO production from resident peritoneal macrophages by in vitro γ-irradiation and its relationship to reactive oxygen intermediates. *Free Radical Bio Med* **22**: 1029–1035

Jenkins DC, Charles IG, Baylis SA, Lelchuk R, Radomski MW and Moncada S (1994) Human colon cancer cell lines show a diverse pattern of nitric oxide synthase gene expression and nitric oxide generation. *Br J Cancer* **70**: 847–849

Jenkins DC, Charles IG, Thomsen LL, Moss DW, Holmes LS, Baylis SA, Rhodes P, Westmore K, Emson PC and Moncada S (1995) Roles of nitric oxide in tumour growth. *Proc Natl Acad Sci USA* **92**: 4392–4396

Kwon NS, Nathan CF, Gilker C, Griffith OW, Matthews DE and Stuehr DJ (1990) L-Citrulline production from L-arginine by macrophage nitric oxide synthase. *J Biol Chem* **265**: 13442–13445

Lams S, Marsden PA, Li JK, Tempst P and Michel T (1992) Endothelial nitric oxide synthase: molecular cloning and characterization of a constitutive enzyme isoform. *Proc Natl Acad Sci USA* **89**: 6348–6352

Laskin DL, Rodriguez del Valle M, Heck DE, Hwang SM, Ohnishi ST, Durham SK, Goller NL and Laskin JD (1995) Hepatic nitric oxide production following acute endotoxemia in rats is mediated by increased inducible nitric oxide synthase gene expression. *Hepatology* **22**: 223–234

Lepoivre M, Boudbid H and Petit J-F (1989) Antiproliferative activity of γ-interferon combined with lipopolysaccharide on murine adenocarcinoma: dependence on an L-arginine metabolism with production of nitrite and citrulline. *Cancer Res* **49**: 1970–1976

Lepoivre M, Chenais B, Yapo A, Lemaire G, Thelander L and Tenu JP (1990) Alterations of ribonucleotide reductase activity following induction of the nitrite-generating pathway in adenocarcinoma cells. *J Biol Chem* **266**: 14143–14149

Lowry OH, Rosebrough NJ, Farr AL and Randall RJ (1951) Protein measurement with the Folin phenol reagent. *J Biol Chem* **193**: 265–275

Moncada S, Palmer MJ and Higgs EA (1991) Nitric oxide: physiology, pathophysiology and pharmacology. *Pharm Rev* **43**: 109–142

Nguyen T, Brunson D, Crespi CL, Penman BW, Wishnok JS and Tannenbaum SR (1992) DNA damage and mutation in human cells exposed to nitric oxide in vitro. *Proc Natl Acad Sci USA* **89**: 3030–3034

Palmer RMJ, Ferrige AG and Moncada S (1987) Nitric oxide release accounts for the biological activity of endothelium-derived relaxing factor. *Nature* **327**: 524–526

Pettengill OS, Bell RH, Kuhlmann ET and Longnecker DS (1993) Derivative of duct like cell lines from a transplantable acinar cell carcinoma of the pancreas. *Am J Pathol* **143**: 292–303

Richards GM (1974) Modifications of the diphenylamine reaction giving increased sensitivity and simplicity in the estimation of DNA. *Anal Biochem* **57**: 369–376

Schneider WC (1957) Determination of nucleic acids in tissues by pentose analysis. In *Methods in Enzymology*, Colowick SP and Kaplan NO (eds), pp. 680–684. Academic Press: New York

Stuehr DJ and Marletta MA (1987) Synthesis of nitrite and nitrate in murine macrophage cell lines. *Cancer Res* **47**: 5590–5594

Stuehr DJ and Nathan C (1989) Nitric oxide: a macrophage product responsible for cytostasis and respiratory inhibition in tumor target cells. *J Exp Med* **169**: 1543–1555

Taniguchi N, Pickett JB and Griffith OW (1993) Oxy radicals and antioxidative responses in cancer: 12th Sapporo Cancer Seminar. *Cancer Res* **53**: 3207–3210

Thomas C, Nijenhuis AM, Dontje B, Daemen T and Scherphof GL (1995) Tumoricidal response of liver macrophages isolated from rats bearing liver metastases of colon adenocarcinoma. *J Leukoc Biol* **57**: 617–623

Weinberg JB, Chapman HA and Hibbs JB (1978) Characterization of the effects of endotoxin on macrophage tumor cell killing. *J Immunol* **121**: 72–80

Xie QW, Cho HJ, Calacay J, Mumford RA, Swiderek KM, Lee TD and Nathan C (1992) Cloning and characterization of inducible nitric oxide synthase from mouse macrophages. *Science* **256**: 225–228

© Cancer Research Campaign 1998